# Single‐institution retrospective review of patients with recurrent glioblastoma treated with bevacizumab in clinical practice

**DOI:** 10.1002/hsr2.114

**Published:** 2019-02-13

**Authors:** Annick Desjardins, James E. Herndon, Frances McSherry, Arliene Ravelo, Eric S. Lipp, Patrick Healy, Katherine B. Peters, John H. Sampson, Dina Randazzo, Nicolas Sommer, Allan H. Friedman, Henry S. Friedman

**Affiliations:** ^1^ The Preston Robert Tisch Brain Tumor Center Duke University Medical Center Durham North Carolina; ^2^ Department of Biostatistics and Bioinformatics Duke University Medical Center Durham North Carolina; ^3^ Duke Cancer Institute Biostatistics Durham North Carolina; ^4^ Health Economics and Outcomes Research US Medical Affairs, Genentech, Inc South San Francisco California

**Keywords:** bevacizumab, real‐world setting, recurrent glioblastoma, survival, treatment patterns

## Abstract

**Background and aims:**

This retrospective review of patients with recurrent glioblastoma treated at the Preston Robert Tisch Brain Tumor Center investigated treatment patterns, survival, and safety with bevacizumab in a real‐world setting.

**Methods:**

Adult patients with glioblastoma who initiated bevacizumab at disease progression between January 1, 2009, and May 14, 2012, were included. A Kaplan‐Meier estimator was used to describe overall survival (OS), progression‐free survival (PFS), and time to greater than or equal to 20% reduction in Karnofsky Performance Status (KPS). The effect of baseline demographic and clinical factors on survival was examined using a Cox proportional hazards model. Adverse event (AE) data were collected.

**Results:**

Seventy‐four patients, with a median age of 59 years, were included in this cohort. Between bevacizumab initiation and first failure, defined as the first disease progression after bevacizumab initiation, biweekly bevacizumab and bevacizumab/irinotecan were the most frequently prescribed regimens. Median duration of bevacizumab treatment until failure was 6.4 months (range, 0.5‐58.7). Median OS and PFS from bevacizumab initiation were 11.1 months (95% confidence interval [CI], 7.3‐13.4) and 6.4 months (95% CI, 3.9‐8.5), respectively. Median time to greater than or equal to 20% reduction in KPS was 29.3 months (95% CI, 13.8‐∞). Lack of corticosteroid usage at the start of bevacizumab therapy was associated with both longer OS and PFS, with a median OS of 13.2 months (95% CI, 8.6‐16.6) in patients who did not initially require corticosteroids versus 7.2 months (95% CI, 4.8‐12.5) in those who did (*P* = 0.0382, log‐rank), while median PFS values were 8.6 months (95% CI, 4.6‐9.7) and 3.7 months (95% CI, 2.7‐6.6), respectively (*P* = 0.0243, log‐rank). Treatment failure occurred in 70 patients; 47 of whom received salvage therapy, and most frequently bevacizumab/carboplatin (7/47; 14.9%). Thirteen patients (18%) experienced a grade 3 AE of special interest for bevacizumab.

**Conclusions:**

Treatment patterns and outcomes for patients with recurrent glioblastoma receiving bevacizumab in a real‐world setting were comparable with those reported in prospective clinical trials.

## 1 INTRODUCTION

Glioblastomas are the most common and most aggressive form of primary brain tumors in adults.^1^ Patients with glioblastoma have a very poor prognosis because of the high propensity for relapse,^2^ with reported median survival times for patients with recurrent disease of just 3 to 9 months.^3^, ^4^ The angiogenic factor vascular endothelial growth factor (VEGF) is expressed at high levels in glioblastoma relative to other cancer types^4^; thus, inhibitors of VEGF have been investigated for the treatment of glioblastoma.^5^

In 2009, the anti‐VEGF monoclonal antibody bevacizumab received accelerated United States Food and Drug Administration (FDA) approval as a single agent for the treatment of recurrent glioblastoma.^6^ Approval of bevacizumab was based on durable objective response rates (ORRs) and 6‐month progression‐free survival (PFS) data obtained from two single‐arm phase 2 studies. The first study evaluated the activity of bevacizumab monotherapy followed by bevacizumab plus irinotecan at disease progression (PD). The 6‐month PFS rate was 29.0%, median overall survival (OS) was 31 weeks, and radiographic response was recorded in 71% and 35% of patients, based on Levin and Macdonald criteria, respectively.^7^ A companion study assessed the efficacy of bevacizumab alone and in combination with irinotecan following PD. In the bevacizumab monotherapy arm, the 6‐month PFS rate was 42.6%, median OS was 9.2 months, and the ORR was 28.2%.^8^ Grade 3/4 adverse events (AEs) across the two studies were mostly nonhematologic and included hypertension and thromboembolic events.^7^, ^8^

Patients enrolled in randomized clinical trials do not always reflect real‐world populations. Real‐world data can provide valuable insights into treatment patterns as well as therapeutic benefits for patients in routine clinical practice. However, there are few real‐world studies evaluating survival outcomes among patients with recurrent glioblastoma. A recent retrospective, online chart‐abstraction study examined patterns of treatment, outcomes, and use of cancer‐related health‐care resource for 503 patients with glioblastoma treated in real‐world clinical practices in the United States, but only 11 patients (2.2%) had recurrent disease.^9^ We retrospectively reviewed treatment patterns, survival, and safety outcomes for patients with recurrent glioblastoma receiving bevacizumab‐containing regimens outside of a clinical protocol at a large specialist brain cancer center in the United States.

## 2 MATERIALS AND METHODS

### 2.1 Study design

This was a retrospective, single‐center study designed to investigate the use of bevacizumab, given at the treating physician's recommendation, in patients with recurrent glioblastoma at the Preston Robert Tisch Brain Tumor Center (PRTBTC), Duke University, Durham, North Carolina. This retrospective study was approved by the institutional review board at Duke University. The “Primary and Recurrent Glioblastoma Registry” (PRoGREss; Pro00027120) was used to identify patients at the PRTBTC with recurrent glioblastoma who were treated with bevacizumab at the time of PD and who initiated bevacizumab therapy between January 1, 2009, and May 14, 2012.

Selected patients were aged greater than or equal to 18 years and had not previously participated in a clinical trial or received bevacizumab. Patients who were alive as of May 15, 2012, signed a consent form permitting the collection of retrospective and prospective data. A waiver or alteration of consent and Health Insurance Portability and Accountability Act (HIPAA) authorization and an Institutional Review Board notification of decedent research were approved by the institutional review board at Duke University to permit the analysis of retrospective data from patients who died before May 15, 2012. The data cut‐off date for the efficacy and safety analyses was April 29, 2014.

The aims of this retrospective review were to describe patterns of treatment, survival outcomes, and toxic effects in patients who received bevacizumab at the time of recurrence and were previously bevacizumab‐naïve. We also explored the associations of baseline demographic and disease characteristics on survival outcomes in these patients.

### 2.2 Assessments

OS was defined as the time from the initiation of bevacizumab until death (OS_bev_). In a separate analysis, OS was defined from the time of glioblastoma diagnosis until death (OS_diag_). For patients alive at the cut‐off date, OS was censored at the last follow‐up visit. PFS was defined as the time from the initiation of bevacizumab until first documentation of PD (as defined by Response Assessment in Neuro‐Oncology [RANO] criteria^10^) or death. For patients alive without PD at the data cut‐off date, PFS was censored at the last follow‐up visit. In analyses evaluating time to a greater than or equal to 20% reduction in Karnofsky Performance Status (KPS), this outcome was defined as the time from initiation of bevacizumab until a KPS reduction of greater than or equal to 20%. For patients without a greater than or equal to 20% KPS reduction either at the time of death or as of the last follow‐up visit for those still alive, this time was censored as of the last KPS assessment date.

Use of bevacizumab in the recurrent setting was defined as patients treated with a bevacizumab‐based regimen after having experienced PD as a glioblastoma patient or previously diagnosed as having a glioma of lower grade. Bevacizumab treatment failure was defined as the first PD following the initiation of bevacizumab treatment; the patient did not have to be receiving bevacizumab at the time of PD.

Toxicity was recorded and graded according to the National Cancer Institute Common Terminology Criteria for Adverse Events (NCI‐CTCAE), version 4.0. The following were predefined as AEs of special interest (AESI) for bevacizumab: new or worsening hypertension (grade ≥3); proteinuria (grade ≥3); gastrointestinal perforation, abscess, or fistulae (any grade); wound‐healing complications (grade ≥3); noncentral nervous system (CNS) hemorrhage (grade ≥3); CNS hemorrhage (any grade); stroke or myocardial infarction (any grade); thromboembolic events (grade ≥3); congestive heart failure (grade ≥3); nongastrointestinal abscesses and fistulae (grade ≥2); and reversible posterior leukoencephalopathy syndrome (any grade).

### 2.3 Statistical analysis

Categorical variables were described with frequency distributions, two‐sided tests were performed, and the SAS software, version 9.4 (SAS Institute), was used for all the analyses. The Kaplan‐Meier estimator was used to describe OS, PFS, and time to greater than or equal to 20% reduction in KPS. The Cox proportional hazards model was used to assess the effect of patient baseline demographic and clinical factors on OS and PFS. Safety was summarized using the maximum NCI‐CTCAE grade experienced for each type of AE.

## 3 RESULTS

### 3.1 Patients

Between January 1, 2009, and May 14, 2012, seventy‐four patients with recurrent glioblastoma were treated with bevacizumab at the PRTBTC: 59 patients (79.7%) initiated a bevacizumab‐based regimen at first progression, following standard radiotherapy and temozolomide first‐line treatment; 12 patients (16.2%) initiated a bevacizumab‐based regimen at second progression; and three patients (4.1%) initiated a bevacizumab‐based regimen after three tumor progressions. The median patient age at the start of bevacizumab therapy was 59 years (range, 22‐88), and most patients had primary (69/74, 93.2%), unifocal (63/74, 85.1%) glioblastoma (Table 1). Just over half of the patients (39/74, 52.7%) had a KPS score of 70 to 80 within the 2 months prior to starting bevacizumab treatment. Prior to the initiation of bevacizumab, 73 patients (98.6%) had received radiotherapy/temozolomide and 31 patients (41.9%) were on corticosteroids.

**Table 1:** Patient baseline characteristics

Characteristic	N = 74, n (%)
Median age, y (range)	59 (22‐88)
Sex	
Male	43 (58.1)
Female	31 (41.9)
Race^b^	
White	70 (94.6)
Black or African American	3 (4.1)
Asian	1 (1.4)
Extent of disease	
Unifocal	63 (85.1)
Multifocal	11 (14.9)
Pathologic glioblastoma status	
Primary	69 (93.2)
Secondary	5 (6.8)
Extent of resection within 60 days of initial glioblastoma diagnosis	
Gross total resection	40 (54.1)
Subtotal resection	26 (35.1)
Biopsy	8 (10.8)
Tumor location	
Temporal lobe	27 (36.5)
Frontal lobe	18 (24.3)
Parietal lobe	14 (18.9)
Multifocal	11 (14.9)
Other location	4 (5.4)
KPS within 2 months prior to starting bevacizumab	
90‐100	20 (27.0)
70‐80	39 (52.7)
≤60	8 (10.8)
Unknown	7 (9.5)
Number of disease progressions prior to starting bevacizumab	
1	59 (79.7)
2	12 (16.2)
3	3 (4.1)
MGMT IHC status	
Negative	13 (17.6)
Positive	12 (16.2)
Test not ordered	49 (66.2)
Prebevacizumab corticosteroid medication	
No	40 (54.1)
Yes	31 (41.9)
Unknown	3 (4.1)
Prebevacizumab antihypertensive medication	
No	43 (58.1)
Yes	31 (41.9)
Medical history^a^	
None relevant	36 (48.6)
Hypertension	28 (37.8)
Gastrointestinal disease, Crohn's disease, ulcerative colitis	10 (13.5)
Cardiac disease	6 (8.1)
Thromboembolic event or pulmonary embolism	6 (8.1)
Stroke	1 (1.4)
Chemotherapy/radiotherapy received prior to initial bevacizumab^a^	
Radiotherapy/temozolomide	73 (98.6)
Temozolomide	68 (91.9)
Carmustine wafers	7 (9.5)
Metronomic temozolomide	7 (9.5)
Lomustine	2 (2.7)
Etoposide	2 (2.7)
Stereotactic radiosurgery	1 (1.4)
Carboplatin/irinotecan	1 (1.4)
Temozolomide/etoposide	1 (1.4)
Radiotherapy	1 (1.4)

Abbreviations: IHC, immunohistochemistry; KPS, Karnofsky Performance Status; MGMT, methylguanine‐DNA methyltransferase.

a Patients may be included in more than one category.

b Self‐reported.

### 3.2 Initial bevacizumab‐containing treatment regimens

The median time between diagnosis of glioblastoma and initiation of bevacizumab treatment was 9 months (range, 1‐45). The most frequently prescribed bevacizumab‐containing regimens before first bevacizumab failure, noting that patients may be included in more than one regimen category, were biweekly bevacizumab (21/74, 28.4%), bevacizumab/irinotecan (21/74, 28.4%), bevacizumab/temozolomide (12/74, 16.2%), bevacizumab/etoposide (11/74, 14.9%), and monthly bevacizumab (8/74, 10.8%) (Table 2). Most patients received a bevacizumab dose of 10 mg/kg. The median bevacizumab treatment duration (including potential stops and restarts) prior to first bevacizumab failure was 6.4 months (range, 0.5‐58.7).

**Table 2:** Bevacizumab‐containing treatment regimens received prior to first treatment failure^a^

Regimen	N = 74, n (%)
Bevacizumab biweekly	21 (28.4)
Bevacizumab/irinotecan	21 (28.4)
Bevacizumab/temozolomide	12 (16.2)
Bevacizumab/etoposide	11 (14.9)
Bevacizumab monthly	8 (10.8)
Bevacizumab/metronomic temozolomide	7 (9.5)
Bevacizumab/carboplatin/irinotecan	4 (5.4)
Stereotactic radiosurgery/bevacizumab/temozolomide	3 (4.1)
Bevacizumab/carboplatin	2 (2.7)
Bevacizumab/imatinib	2 (2.7)
Bevacizumab/irinotecan/sorafenib	1 (1.4)
Bevacizumab/temozolomide/imatinib	1 (1.4)
Stereotactic radiosurgery/bevacizumab/irinotecan	1 (1.4)

a Patients may be included in more than one category.

### 3.3 Survival

**Figure 1:**
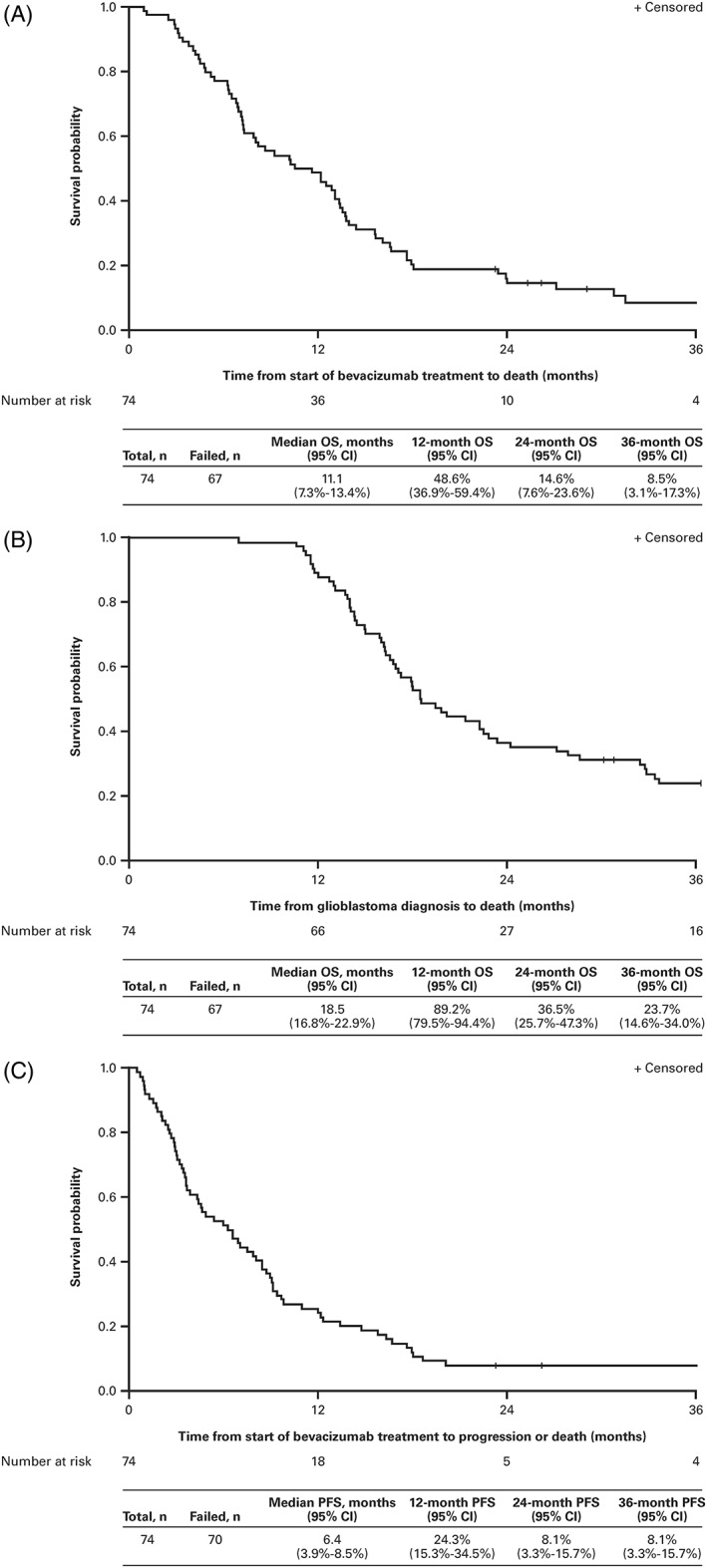
Kaplan‐Meier curves of: A, overall survival from initiation of bevacizumab; B, overall survival from glioblastoma diagnosis; and C, progression‐free survival from initiation of bevacizumab. CI indicates confidence interval; OS, overall survival; PFS, progression‐free survival

The median duration of follow‐up at the time of data cut off was 43.8 months (95% confidence interval [CI], 25.3‐58.0). At that time, 70 patients (94.6%) had progressed and 67 patients (90.5%) had died. The median OS_bev_ and OS_diag_ were 11.1 months (95% CI, 7.3‐13.4) and 18.5 months (95% CI, 16.8‐22.9), respectively (Figure 1A and 1B). The percentage of patients alive at 12, 24, and 36 months after initiation of bevacizumab was 48.6%, 14.6%, and 8.5%, respectively. Median OS_bev_ for patients initially treated with bevacizumab at first recurrence was 10.2 months (95% CI, 7.2‐13.4) versus 13.1 months (95% CI, 6.3‐18.1) for those who were initially treated with bevacizumab at a later recurrence (*P* = 0.2226, log‐rank). The median PFS for all patients was 6.4 months (95% CI, 3.9‐8.5), with 24.3%, 8.1%, and 8.1% of patients alive without PD at 12, 24, and 36 months, respectively (Figure 1C).

### 3.4 Performance status

**Figure 2:**
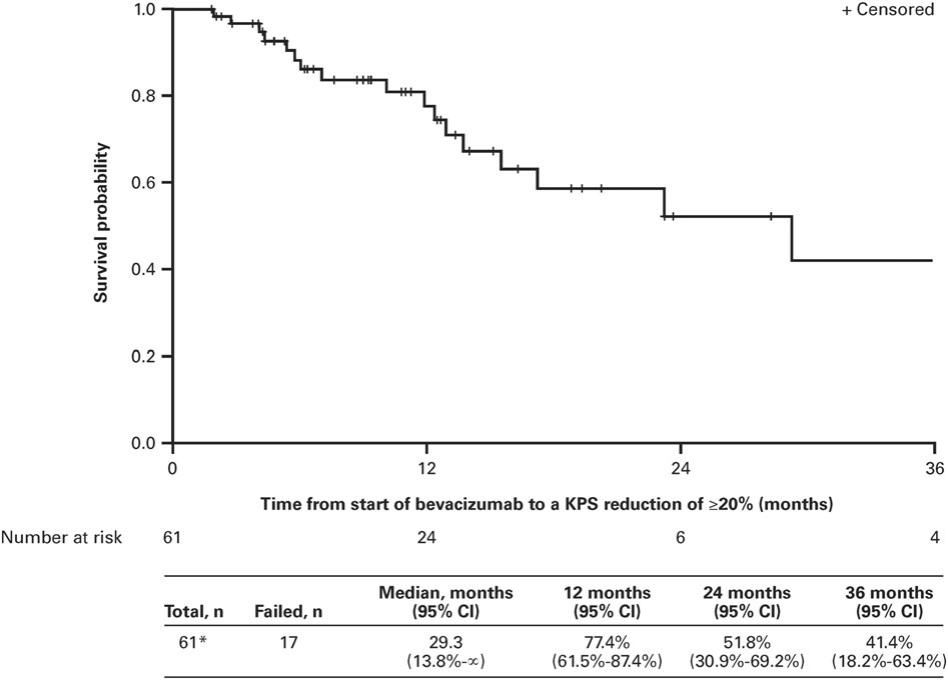
Time to a greater than or equal to 20% reduction in KPS from initiation of bevacizumab‐based treatment. *Thirteen patients were missing their baseline and/or their post‐baseline KPS. CI indicates confidence interval; KPS, Karnofsky performance status

Of the 61 patients with a baseline KPS available within 2 months prior to starting bevacizumab and at least one follow‐up KPS assessment, 17 patients (27.9%) experienced a greater than or equal to 20% reduction in KPS from bevacizumab initiation (Figure 2). The median time to greater than or equal to 20% reduction in KPS from bevacizumab initiation was 29.3 months (95% CI, 13.8‐∞), and the proportion of patients without a KPS reduction of greater than or equal to 20% from bevacizumab initiation was 77.4%, 51.8%, and 41.4% at 12, 24, and 36 months, respectively.

### 3.5 Corticosteroid use during bevacizumab treatment

Overall, 31/74 patients (41.9%) were receiving corticosteroids at the time of bevacizumab initiation. Of these, 17 patients (54.8%) discontinued corticosteroids during bevacizumab therapy and one patient (3.2%) discontinued corticosteroids while not receiving bevacizumab. Forty of the 74 patients (54.1%) were not taking corticosteroids at the start of bevacizumab treatment. Of these, 16 (40.0%) started corticosteroid therapy during bevacizumab treatment with a median time to corticosteroid initiation of 70.5 days (range, 1‐552), two patients (5.0%) started corticosteroids after discontinuation of bevacizumab, and 22 patients (55.0%) never received steroids at any time after bevacizumab was initiated.

Lack of corticosteroid usage at the start of bevacizumab therapy was associated with both longer OS and PFS. Median OS was 13.2 months (95% CI, 8.6‐16.6) in patients who did not initially require corticosteroids versus 7.2 months (95% CI, 4.8‐12.5) in those who did (*P* = 0.0382, log‐rank). Corresponding median PFS values were 8.6 months (95% CI, 4.6‐9.7) and 3.7 months (95% CI, 2.7‐6.6), respectively (*P* = 0.0243, log‐rank).

### 3.6 Safety

AEs reported at a cumulative incidence of greater than or equal to 10% across all grades during bevacizumab therapy (including salvage bevacizumab treatment) are displayed in Table 3. However, assessment of attribution to bevacizumab or other therapy was not completed. The maximum grade AE experienced by most patients (41/74, 55%) was grade 3; seven patients (9%) experienced a total of 10 grade 4 AEs. The most frequently reported grade 3/4 AEs were white blood cell count decreased (12/74, 16%), fatigue (10/74, 14%; grade 3 only), and neutrophil count decreased (9/74, 12%). There were no deaths due to AEs.

**Table 3:** Adverse events experienced during any bevacizumab treatment (prior to first bevacizumab failure or during salvage bevacizumab treatment) occurring at a cumulative incidence of greater than or equal to 10% across all grades

	Grade 1, n (%)	Grade 2, n (%)	Grade 3, n (%)	Grade 4, n (%)
Hematologic adverse events				
Blood and lymphatic system disorders				
Anemia	34 (46)	10 (14)	1 (1)	0
Investigations				
Platelet count decreased	34 (46)	9 (12)	5 (7)	0
White blood cell count decreased	13 (18)	15 (20)	10 (14)	2 (3)
Neutrophil count decreased	6 (8)	8 (11)	6 (8)	3 (4)
Nonhematologic adverse events				
Investigations				
Alanine aminotransferase increased	18 (24)	3 (4)	2 (3)	0
Alkaline phosphatase increased	11 (15)	1 (1)	0	0
Aspartate aminotransferase increased	16 (22)	1 (1)	2 (3)	0
Gastrointestinal disorders				
Nausea	33 (45)	7 (9)	0	0
Diarrhea	26 (35)	3 (4)	2 (3)	0
Constipation	18 (24)	4 (5)	0	0
Vomiting	11 (15)	4 (5)	0	0
Dysphagia	3 (4)	5 (7)	1 (1)	0
Abdominal pain	2 (3)	5 (7)	0	0
General disorders and administration site conditions				
Fatigue	19 (26)	23 (31)	10 (14)	0
Edema (limbs)	11 (15)	4 (5)	0	0
Pain	5 (7)	4 (5)	0	0
Infections and infestations				
Urinary tract infection	1 (1)	8 (11)	0	0
Upper respiratory infection	4 (5)	5 (7)	0	0
Injury, poisoning, and procedural complications				
Fall	13 (18)	4 (5)	1 (1)	0
Metabolism and nutrition disorders				
Hyperglycemia	25 (34)	20 (27)	3 (4)	0
Hypocalcemia	19 (26)	5 (7)	0	0
Hypokalemia	21 (28)	0	3 (4)	0
Hyponatremia	13 (18)	0	4 (5)	0
Anorexia	6 (8)	5 (7)	0	0
Hypoglycemia	5 (7)	2 (3)	0	1 (1)
Musculoskeletal and connective tissue disorders				
Back pain	9 (12)	1 (1)	0	0
Generalized muscle weakness	1 (1)	9 (12)	4 (5)	0
Nervous system disorders				
Headache	26 (35)	10 (14)	4 (5)	0
Memory impairment	16 (22)	9 (12)	0	0
Dysphasia	5 (7)	14 (19)	5 (7)	0
Seizure	9 (12)	10 (14)	2 (3)	1 (1)
Paresthesia	13 (18)	1 (1)	0	0
Cognitive disturbance	3 (4)	9 (12)	3 (4)	0
Ataxia	5 (7)	3 (4)	1 (1)	0
Imbalance	4 (5)	5 (7)	0	0
Gait disturbance	3 (4)	4 (5)	2 (3)	0
Right hemiparesis	2 (3)	4 (5)	2 (3)	0
Dizziness	8 (11)	0	0	0
Tremor	4 (5)	4 (5)	0	0
Psychiatric disorders				
Confusion	4 (5)	6 (8)	1 (1)	0
Depression	6 (8)	2 (3)	0	0
Anxiety	4 (5)	4 (5)	0	0
Renal and urinary disorders				
Proteinuria	7 (9)	12 (16)	1 (1)	0
Urinary incontinence	9 (12)	3 (4)	1 (1)	0
Respiratory, thoracic, and mediastinal disorders				
Epistaxis	11 (15)	0	1 (1)	0
Cough	8 (11)	2 (3)	0	0
Dyspnea	6 (8)	3 (4)	1 (1)	1 (1)
Vascular disorders				
Hypertension	2 (3)	0	7 (9)	0
Thromboembolic event	0	5 (7)	3 (4)	0

Note that patients can be included more than once for different adverse events (AEs) and more than once for the same AE at different grades.

During bevacizumab treatment, 15 grade 3 AESIs occurred in 13 patients (18%): myocardial infarction (n = 1), diverticular abscess (n = 1), ejection fraction decreased (n = 1), proteinuria (n = 1), bronchopulmonary hemorrhage (n = 1), hypertension (n = 7, 9%), and thromboembolic event (n = 3, 4%). There were no grade 4 AESIs.

### 3.7 Patterns of treatment until bevacizumab failure

**Figure 3:**
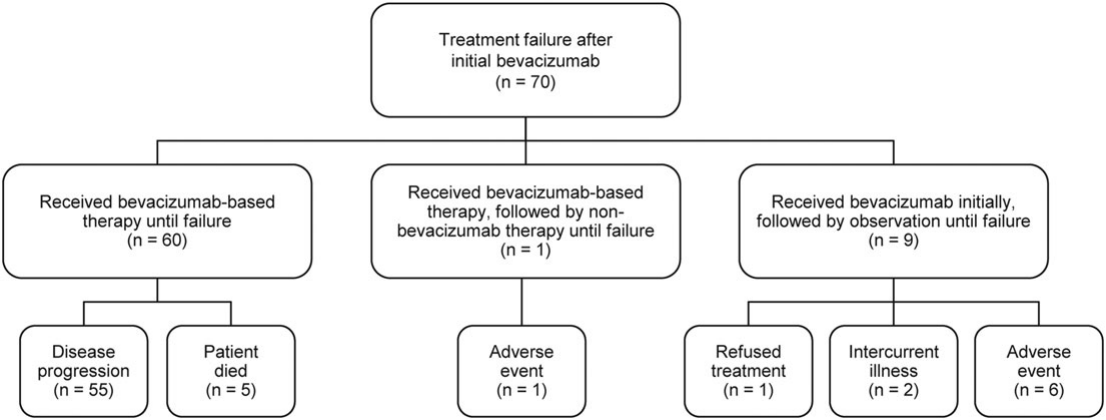
Patterns of treatment until bevacizumab failure

Treatment failure after initial bevacizumab occurred in 70 patients. Sixty of these patients (85.7%) received bevacizumab‐based therapy until failure, including 55 patients (78.6%) who had PD and five patients (7.1%) who died (Figure 3 and Table S1). Nine of the 70 patients (12.9%) were off all therapy at the time of failure. Six of these patients stopped the bevacizumab‐based regimen because of AEs, two patients had intercurrent illnesses, and one patient refused further treatment. One of the 70 patients (1.4%) received bevacizumab‐based therapy followed by nonbevacizumab therapy prior to failure.

After initial bevacizumab treatment failure, 47 patients received salvage therapy (Table S2). Overall, 41 of the 47 patients (87.2%) received a bevacizumab‐based regimen as their first salvage regimen; 38 of these 41 patients remained on a bevacizumab‐based regimen until they died. The most frequently prescribed first salvage regimens were bevacizumab/carboplatin (7/47, 14.9%) and bevacizumab/irinotecan and bevacizumab/metronomic temozolomide (5/47, 10.6% each).

## 4 DISCUSSION

Because of the heterogeneous nature of glioblastoma, there is currently no standard treatment for patients with recurrent disease.^2^ Single‐agent bevacizumab or bevacizumab in combination with chemotherapy are accepted recurrence therapies in treatment practice guidelines.^11^ We retrospectively reviewed data for patients with recurrent glioblastoma who initiated a bevacizumab‐containing regimen at the PRTBTC between January 1, 2009, and May 14, 2012, to assess treatment patterns and the efficacy and safety of bevacizumab administered outside the context of a clinical trial.

Prior to first failure after initiation of bevacizumab, the most frequently prescribed bevacizumab‐containing regimens were biweekly bevacizumab, bevacizumab/irinotecan, bevacizumab/temozolomide, bevacizumab/etoposide, and monthly bevacizumab. Across all treatment regimens, the median OS_bev_ and PFS were 11.1 months (95% CI, 7.3‐13.4) and 6.4 months (95% CI, 3.9‐8.5), respectively. These data, obtained in a real‐world treatment setting, compare favorably with the results of the pivotal phase 2 trials of bevacizumab in recurrent glioblastoma, which reported median OS_bev_ of 31 weeks^7^ and 9.2 months,^8^ and median PFS of 16 weeks^7^ and 4.2 months.^8^ Our findings are also in agreement with a median OS of 9.3 months and median time to progression of 6.1 months recorded in a meta‐analysis of 15 studies of patients with recurrent glioblastoma receiving bevacizumab.^12^ It is noteworthy that our patients were initiated on treatment before the publication of the BELOB trial,^13^ and thus, the prescribed regimen would most probably be different if the analyses were repeated today.

Limited real‐world data have been published on treatment patterns and survival outcomes in patients with recurrent glioblastoma receiving bevacizumab. In a retrospective population‐based Canadian study, in which the majority of patients (113/160, 70.6%) had recurrent glioblastoma, administration of bevacizumab with or without lomustine or etoposide resulted in a median OS of 7 months and a median PFS of 4 months.^14^ Chen et al analyzed the efficacy and safety of bevacizumab given alone or in combination with irinotecan versus nonbevacizumab‐containing therapy for 159 bevacizumab‐naïve patients with recurrent glioblastoma treated in community‐based practices in the United States.^15^ After adjustment for confounders, multivariate Cox models of second‐line bevacizumab monotherapy showed a statistically nonsignificant increase in OS compared with nonbevacizumab treatment (hazard ratio [HR], 0.56 [95% CI, 0.31‐1.03]), and patients receiving bevacizumab combination regimens had longer OS than patients not receiving any bevacizumab (HR, 0.34 [95% CI, 0.21‐0.68]). There was no difference in PFS between bevacizumab monotherapy patients and those receiving nonbevacizumab treatment (HR, 0.98 [95% CI, 0.50‐1.92]), and patients receiving bevacizumab combination regimens showed a statistically nonsignificant increase in PFS (HR, 0.52 [95% CI, 0.27‐1.01]) compared with patients on nonbevacizumab‐based treatment. In contrast, the addition of bevacizumab to lomustine did not extend OS versus lomustine alone in 437 patients with recurrent glioblastoma enrolled in the EORTC 26101 clinical study (HR, 0.95 [95% CI, 0.74‐1.21]), despite prolonged PFS (HR, 0.49 [95% CI, 0.39‐0.61]).^16^

No specific patient baseline or disease characteristics were associated with the improved survival outcomes seen in the analysis by Chen and colleagues.^15^ However, we observed prolonged OS and PFS in patients who did not require corticosteroids at the start of bevacizumab therapy, as reported previously in pooled analyses of patients with recurrent glioblastoma enrolled in clinical trials.^17^, ^18^ No statistically significant difference in median OS_bev_ was observed for patients receiving bevacizumab at first versus later recurrence, which is in agreement with previously published data for patients with recurrent glioblastoma treated in clinical practice stating that timing of bevacizumab treatment does not impact survival.^19^ This is also likely to be impacted by the fact that patients needing bevacizumab at first recurrence are those with a larger tumor or a more rapid disease progression than patients who initiate it later in their disease course.

Results of a large population‐based Surveillance, Epidemiology, and End Results (SEER) analysis demonstrated improved OS in patients diagnosed with glioblastoma between 2010 and 2012 (postbevacizumab approval cohort, n = 6753) compared with those diagnosed between 2006 and 2008 (prebevacizumab approval cohort, n = 6120).^20^ OS rates at 1 and 2 years postdiagnosis were significantly higher for patients in the postbevacizumab cohort (44% and 21%, respectively) versus those in the prebevacizumab cohort (40% and 19%, respectively; *P* < 0.01). After adjusting for confounding factors, the HR for death remained significantly lower in the postbevacizumab cohort (HR, 0.91 [95% CI, 0.87‐0.96]; *P* < 0.01). Although the cause of this survival improvement cannot be proven in a retrospective analysis, the authors conclude that its timing may indicate a potential benefit of bevacizumab in recurrent glioblastoma.

Bevacizumab was well tolerated in our study, with decreased white blood cell count, fatigue and decreased neutrophil count being the most frequently reported grade 3/4 AEs. AESIs for bevacizumab such as hypertension (9%) and thromboembolic events (4%) were reported at a similar rate to the pivotal phase 2 clinical studies of bevacizumab.^7^, ^8^

Our study is limited by its retrospective nature, the inclusion of patients from only a single center, and the lack of data on the molecular genetics of the tumors. The wide variety of bevacizumab‐containing regimens received by the patients, and the fact that patient‐specific treatment was not mandated by a study protocol, also limit the findings. However, in terms of survival, corticosteroid usage, and baseline KPS, our results compare favorably with those published in prospective clinical trials, which may not always accurately reflect real‐world populations.

Taken together, these data indicate an important role for bevacizumab as part of the treatment modality for recurrent glioblastoma. Bevacizumab‐based regimens were the most commonly used regimens both prior to, and following, first bevacizumab failure. Patient outcomes in this real‐world setting were comparable with those reported in prospective clinical trials, and bevacizumab was generally well tolerated.

## FUNDING

The study was funded by Genentech, Inc. Genentech, Inc was involved in the design, writing of the report, and in the decision to submit the report for publication.

## CONFLICTS OF INTEREST

Annick Desjardins has received grants or research support from Genentech, PTC Therapeutics, Celldex, Triphase Research and Development Corp, Eli Lilly and Co, Eisai, Symphogen A/S, Pfizer, and Orbus Therapeutic and is an advisory board member for Genentech; Arliene Ravelo is an employee of Genentech and owns stock options in Roche; Nicolas Sommer is an employee of Genentech and owns stock options in Roche; and the remaining authors have no conflicts of interest to declare.

## AUTHOR CONTRIBUTIONS

Conceptualization: Annick Desjardins, James E. Herndon II, Arliene Ravelo

Data curation: Eric S. Lipp, Patrick Healy

Formal analysis: Annick Desjardins, James E. Herndon II, Frances McSherry, Patrick Healy

Funding acquisition: Annick Desjardins, Arliene Ravelo, Nicolas Sommer

Resources: Annick Desjardins, Eric S. Lipp, Katherine B. Peters, Dina Randazzo, Allan H. Friedman, Henry S. Friedman

Writing—original draft preparation: Annick Desjardins, James E. Herndon II, Frances McSherry, Arliene Ravelo

Writing—review and editing: Annick Desjardins, James E. Herndon II, Frances McSherry, Arliene Ravelo, Eric S. Lipp, Patrick Healy, Katherine B. Peters, John H. Sampson, Dina Randazzo, Nicolas Sommer, Allan H. Friedman, Henry S. Friedman

Annick Desjardins, James E. Herndon II, Eric Lipp, and Frances McSherry had full access to all of the data in this study and take complete responsibility for the integrity of the data and the accuracy of the data analysis.

## Supporting information

Table S1. Reasons for Stopping Last Bevacizumab‐Based Regimen Prior to First Failure Among the 70 Patients With Bevacizumab Treatment Failure
Table S2. First Salvage Treatment (Patients With Bevacizumab Treatment Failure Only)
