# Supplementary material for: Single‐institution retrospective review of patients with recurrent glioblastoma treated with bevacizumab in clinical practice
Source: Health Sci Rep. 2019 Feb 13;2(4):e114. doi: 10.1002/hsr2.114 (PMC6482327; doi:10.1002/hsr2.114)
Supplement: Supplementary file 1 — Table S1. Reasons for Stopping Last Bevacizumab‐Based Regimen Prior to First Failure Among the 70 Patients With Bevacizumab Treatment Failure Table S2. First Salvage Treatment (Patients With Bevacizumab Treatment Failure Only) [file HSR2-2-e114-s001.doc]

**Supporting Information**

**Supporting Table S1.** Reasons for Stopping Last Bevacizumab-Based Regimen Prior to First Failure Among the 70 Patients With Bevacizumab Treatment Failure

| Treatment Pattern Between Initiation of Bevacizumab-Based Regimen and First Failure | Reason for Stopping | n (%) |
| --- | --- | --- |
| Bevacizumab-based treatment until failure | Disease progression | 55 (78.6) |
|  | Death | 5 (7.1) |
| Bevacizumab-based treatment followed by non-bevacizumab treatment until failure | Adverse event | 1 (1.4) |
| Bevacizumab-based treatment followed by observation until failure | Adverse event | 6 (8.6) |
|  | Intercurrent illness | 2 (2.9) |
|  | Refused further treatment | 1 (1.4) |

**Supporting Table S2. First Salvage Treatment (Patients With Bevacizumab Treatment Failure Only)**

| Treatment Regimen, n (%) | N = 47 |
| --- | --- |
| Bevacizumab-based regimen |  |
| Bevacizumab/irinotecan/vorinostat | 1 (2.1) |
| Bevacizumab/buparlisib | 1 (2.1) |
| Bevacizumab/lomustine | 4 (8.5) |
| Bevacizumab/irinotecan | 5 (10.6) |
| Bevacizumab/irinotecan/temozolomide | 2 (4.3) |
| Bevacizumab/carboplatin | 7 (14.9) |
| Bevacizumab/carboplatin/irinotecan | 2 (4.3) |
| Bevacizumab/imatinib/hydroxycarbamide | 2 (4.3) |
| Bevacizumab/metronomic temozolomide | 5 (10.6) |
| Bevacizumab/temozolomide | 1 (2.1) |
| Bevacizumab/temozolomide/etoposide | 2 (4.3) |
| Bevacizumab/etoposide | 4 (8.5) |
| Bevacizumab/etoposide/sirolimus | 1 (2.1) |
| Vorinostat/bevacizumab/temozolomide | 1 (2.1) |
| Stereotactic radiosurgery/bevacizumab | 1 (2.1) |
| Stereotactic radiosurgery/bevacizumab/irinotecan | 1 (2.1) |
| Stereotactic radiosurgery/bevacizumab/etoposide | 1 (2.1) |
| Non-bevacizumab-based regimen |  |
| Irinotecan/panitumumab | 1 (2.1) |
| Carboplatin/irinotecan | 1 (2.1) |
| Stereotactic radiosurgery | 1 (2.1) |
| Metronomic temozolomide | 2 (4.3) |
| CC-122 | 1 (2.1) |
